# Thymoquinone, as an anticancer molecule: from basic research to clinical investigation

**DOI:** 10.18632/oncotarget.17206

**Published:** 2017-04-18

**Authors:** Md. Asaduzzaman Khan, Mousumi Tania, Shangyi Fu, Junjiang Fu

**Affiliations:** ^1^ Key Laboratory of Epigenetics and Oncology, The Research Center for Preclinical Medicine, Southwest Medical University, Luzhou, Sichuan, China; ^2^ Faculty of Applied Sciences, Ton Duc Thang University, Ho Chi Minh City, Vietnam; ^3^ Division of Computer Aided Drug Design, The Red-Green Computing Centre, Dhaka, Bangladesh; ^4^ The Honors College, University of Houston, Houston, Texas, United States

**Keywords:** Nigella sativa, thymoquinone, anticancer, mouse model, clinical studies

## Abstract

Thymoquinone is an anticancer phytochemical commonly found in black cumin. In this review, we discuss the potential of thymoquinone as anticancer molecule, its mechanism of action and future usage in clinical applications. Thymoquinone exhibits anticancer activity via numerous mechanisms of action, specifically by showing selective antioxidant and oxidant activity, interfering with DNA structure, affecting carcinogenic signaling molecules/pathways and immunomodulation. *In vitro* activity of thymoquinone has been further implicated in animal models of cancer; however, no clinical application has been proven yet. This is the optimum time to focus on clinical trials for developing thymoquinone as a future drug in cancer therapeutics.

## INTRODUCTION

Every year, millions of people are diagnosed with cancer, which is the second leading cause of death worldwide after myocardial infarction. Fortunately, the number of cancer survivors is increasing, mainly due to advances in early detection and new treatment strategies. It has been reported that more than 15.5 million Americans with a history of cancer are alive by January 2016 [1]. However, in many regions in the world, including East Asian countries, cancer is still the major public health problem with increasing incidence and mortality rate [2]. As for now, chemotherapy is one of the most common treatment option in cancer therapy, which continues to increase the amount of anticancer drugs used for treatment; even so, most people use a combination of treatments, such as surgery with chemotherapy and radiation therapy.

**Figure 1:**
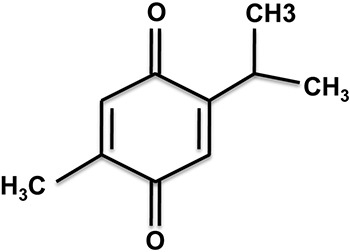
The molecular structure of thymoquinone (Chemical name: 2-Isopropyl-5-methylbenzo-1,4-quinone)

Unfortunately, chemotherapeutic agents create many adverse side effects. Currently, there is a trend in searching for anticancer chemicals in natural sources, as natural products are usually thought to be less toxic and produce minimal side effects. Drugs from natural sources have been used traditionally for thousands of years in various parts of the world. Scientists have targeted many traditional or folk medicines in parallel of modern medicine to identify and extract active ingredients for the drug development. Thymoquinone (2-methyl-5-isopropyl-1,4-benzoquinone) (Figure 1) is a phytochemical compound found in black cumin (*Nigella sativa*) with a long history of medicinal use [3, 4]. The black cumin seeds have a notable history in traditional medicine practices mainly in South and South-eastern Asia, Arab, Africa and Mediterranean regions. In ancient Egypt, Greece and Turkey, black cumin seeds were often used to treat a number of diseases and ailments [3–6]. Both seeds and oil from *Nigella sativa* plants are used in medicinal purposes, and they are known for their anticancer, antidiabetic, antihypertensive, antimicrobial, analgesic, immunomodulatory, anti-inflammatory, spasmolytic, hepato-protective, renal-protective, gastro-protective, bronchodilative and antioxidant activities [4–6]. Its versatile healing abilities have given the black cumin seed its name ‘Panacea’ (in Latin, meaning ‘cure all’), and ‘Habbah Sawda’ or ‘Habbat el Baraka’ (in Arabic, translated as ‘Seeds of blessing)’. It is also known as ‘Kalo jeera’ (in Bangladesh), ‘Kalonji’ (in India) and ‘hēi zhǒng cǎo’ (in China). Black cumin seeds and oil are also known as ‘Prophetic medicine’, as the Islamic prophet has deemed its high potential as medicine [4–7].

In recent years, many scientific studies have revealed the anticancer potential of thymoquinone, however, there is no clinical application yet. In this review, we discuss the potential of thymoquinone as anticancer molecule, and its mechanism of action and future usage in clinical applications.

### Thymoquinone in modern anticancer research: efficacy and mechanisms of action

**Figure 2:**
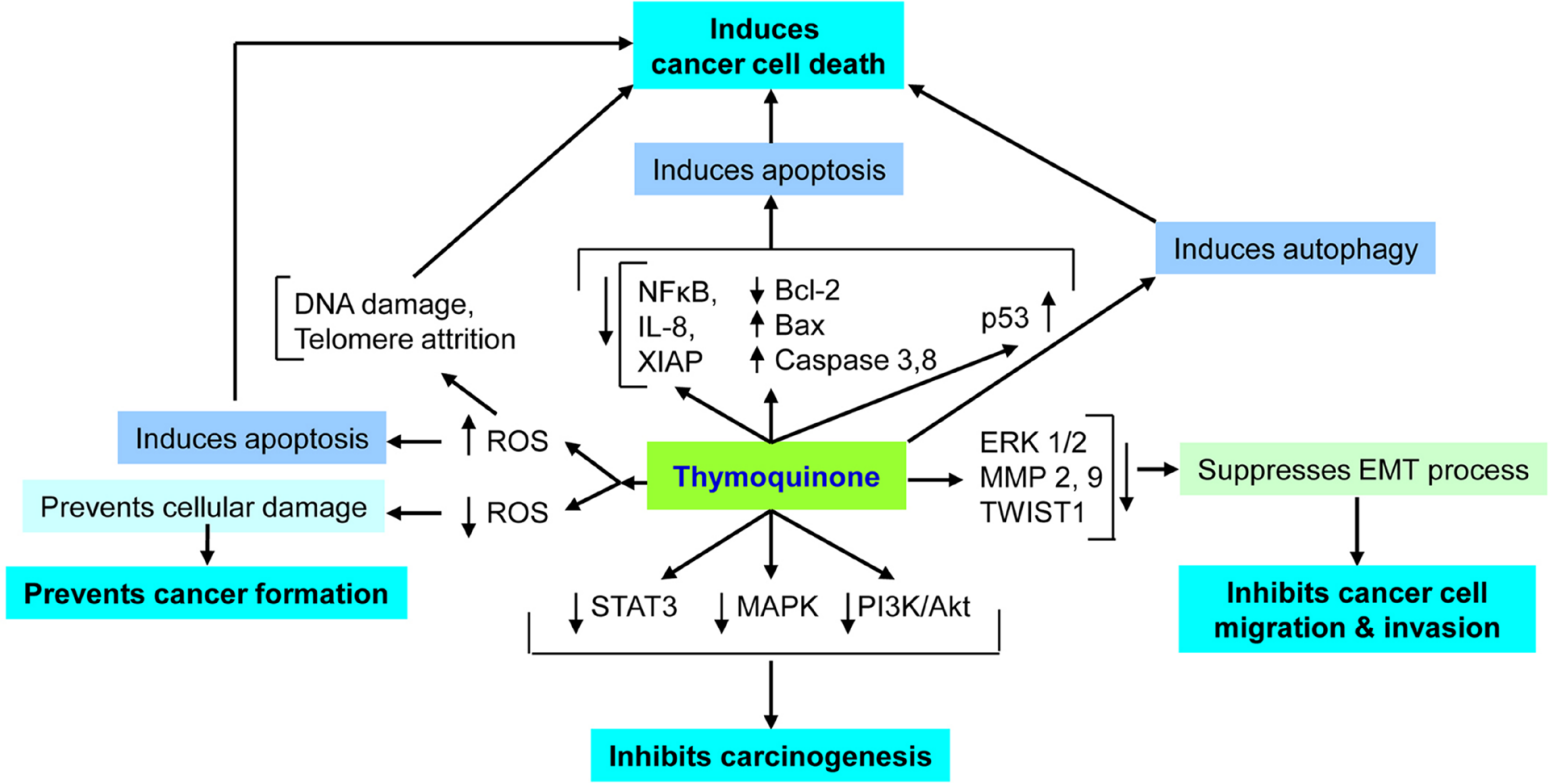
Important mechanisms of thymoquinone's anticancer action Thymoquionone induces apoptosis in cancer cells via generating reactive oxygen species (ROS), DNA damage, telomeric attrition, immunomodulation, regulating signaling pathways and autophagy induction. Thymoquinone also regulates epitheilial to mesenchymal transition (EMT) and inhibits cancer metastasis. In non-cancer cells, thymoquione shows anti-oxidant activities and chemopreventive activity.

Thymoquinone was identified and quantified in black cumin seed oils by Ghosheh *et al*., [8] with other compounds named dithymoquinone, thymohydroquinone and thymol. Over a decade ago, some scientists became interested in anticancer activities of thymoquinone. Since then, a number of studies have been carried out to evaluate the anticancer or chemopreventive role and mechanism of action of thymoquinone in different cancer cell lines and animal models of different cancer types (Table 1, Figure 2).

**Table 1:** Thymoquinone's action against different cancers

Cancer types	Cell lines	Animal model	Mechanism of action of thymoquinone	Ref.
Acute lymphoblastic leukemia	CEM-ss		Generates ROS and HSP70, down-regulates Bcl-2, up-regulates Bax, activates caspase 3, 8 for inducing apoptosis	[30]
Bladder cancer	T24		Attenuates mTOR activity, and inhibits PI3K/Akt signaling	[52, 53]
Breast cancer	MDA-MB-468, T47D		Interferes with PI3K/Akt signaling and promotes G(1) arrest	[27]
	MCF-7		Up-regulates p53	[31]
	BT549		Down-regulates TWIST1 and EMT	[51]
		Mouse	Inhibits NF-κB; Down-regulates p38 MAPK via the generation of ROS; inhibits TWIST1 expression and controls cancer cell metastasis by regulating EMT	[51, 58, 65]
Cervical cancer	HeLa		Inhibits serine/threonine kinase Plk1	[21]
Colon cancer	HCT116		Induces apoptosis by up-regulating Bax and inhibiting Bcl-2, as well as activation of caspases -9, -7 and -3 and induction of PARP cleavage; blocks STAT3 signaling via inhibition of JAK2- and Src-mediated phosphorylation of EGFR tyrosine kinase.	[25]
	CPT-11-R LoVo		Induces caspase-independent autophagy	[43]
		Rat	Exert oxidative stress	[55]
		Mouse	Delays the growth of tumor, reduces tumor cell invasion and also increases apoptosis	[56]
Colorectal cancer	HCT116w, DLD-1, HT29		Binds to oncogene PAK1, changes its conformation and scaffold function, and interferes with RAF/MEK/ERK1/2 pathway and controls cancer cell growth	[47]
Cholangio-carcinoma	TFK-1, HuCCT1		Down-regulates PI3K/Akt and NF-κB, and their regulated gene products, such as p-AKT, p65, XIAP, Bcl-2, COX-2 and VEGF	[46]
Familial adenomatous polyposis		Mouse	Interferes with polyp progression through induction of tumor-cell specific apoptosis and by modulating Wnt signaling through the activation of GSK-3β	[63]
Gastric cancer	HGC27, BGC823, SGC7901		Inhibits STAT3 phosphorylation, associated with reduction in JAK2 and c-Src activity, as well as Bcl-2, cyclin D, survivin, and VEGF	[24]
		Mouse	Down-regulates STAT3	[24]
Glioblastoma	M059K, M059J		Induces DNA damage, telomere attrition by inhibiting telomerase and cell death	[19]
	U-87, CCF-STTG1		Down-regulates FAK, associated with a reduction of ERK phosphorylation as well MMP-2 and MMP-9 secretion, and consequently inhibits cell migration and invasion	[48]
Hepatic carcinoma	HepG2		Stimulates expression of pro-apoptotic Bcl-xS and TRAIL death receptors, and inhibits expression of the anti-apoptotic gene Bcl-2, as well as inhibits NF-κB and IL-8 and stimulates apoptosis	[45]
		Rat	Decreases the expression of antioxidant enzymes, such as, glutathione peroxidase, glutathione-s-transferase and catalase; regulates G1/S phase cell cycle transition	[64]
Lung cancer	A549		Reduces ERK1/2 phosphorylation and controls proliferation and migration	[50]
Multiple myeloma	U266, RPMI8226		Inhibits IL-6-inducible STAT3 phosphorylation, which is correlated with the inhibition of c-Src and JAK2 activation. Also inhibits the expression of STAT3-regulated gene products, D1, Bcl-2, Bcl-xL, survivin, Mcl-1 and VEGF, which ultimately induces apoptosis	[23, 38, 93]
Murine Leukemia	WEHI-3	Mouse	Increases early apoptosis through the up-regulation of Bcl-2, and down-regulation of Bax.	[68]
Myeloid leukemia	KBM-5		Suppresses TNF-α-induced NF-κB activation, and consequently inhibits the activation of I-κB alpha kinase, I-κB alpha phosphorylation, I-κB alpha degradation, p65 phosphorylation, p65 nuclear translocation, and the NF-κB -dependent reporter gene expression; Also down-regulates the expression of NF-κB -regulated antiapoptotic gene products like IAP1, IAP2, XIAP Bcl-2, Bcl-xL, and surviving; proliferative gene products like cyclin D1, cyclooxygenase-2, and c-Myc, and angiogenic gene products MMP-9 and VEGF	[35]
Oral cancer	T28		Down-regulates proliferation activator p38 MAPK	[26]
Osteosarcoma	MG63		Generates ROS to induce oxidative damage and apoptosis	[11, 70]
Prostate Cancer	LNCaP		Antioxidant activity controls cancer cell growth	[9]
	DU145, PC-3, LNCaP		Inhibits DNA synthesis and proliferation	[20]
Pancreatic cancer	FG/COLO357, CD18/HPAF		Down-regulates MUC4 expression through the proteasomal pathway and induces apoptosis by the activation of JNK) and p38 MAPK pathways	[17]
		Mouse	Down-regulates MMP-9, XIAP	[59]
Squamous cell carcinoma		Mouse	Inhibits cell proliferation and induces apoptosis by inhibiting Akt and JNK phosphorylations	[62]

### Antioxidant activity of thymoquinone

Initial experimental studies suggested that the antioxidant activity of thymoquinone is responsible for its chemopreventive activities; however, some other studies reported thymoquinone induce apoptosis in cancer cells by exerting oxidative damage [9–13]. An interesting study reported that thymoquinone is actually a potent apoptosis inducer in cancer cells, but it exerts antiapoptotic effect through attenuating oxidative stress in other types of cell injury [14]. Another hypothesis states that thymoquinone acts as an antioxidant at lower concentrations and a prooxidant at higher concentrations [15]. The antioxidant, antiproliferative and proapoptotic activity of thymoquinone was further explained by Cecarini *et al*. [16], demonstrating that thymoquinone induces selective proteasome inhibition, which could be implicated in the induction of apoptosis in cancer cells. Further study by Torres *et al*. [17] revealed that thymoquinone down-regulates glycoprotein mucin 4 (MUC4) expression through the proteasomal pathway and induces apoptosis in pancreatic cancer cells by activating c-Jun NH(2)-terminal kinase (JNK) and p38 mitogen-activated protein kinase (MAPK) pathways. Usually, MUC4 is aberrantly expressed in pancreatic cancer, and contributes to the regulation of cellular differentiation, proliferation, metastasis and chemoresistance.

### Thymoquinone interferes with DNA structure and synthesis

Thymoquinone also acts via interference with DNA structure. It targets cellular copper, which is present in the chromatin and is closely associated with DNA base guanine, and causes oxidative breakage to DNA and consequent cancer cell death [15]. Thymoquinone can possibly act as a G-quadruplex DNA stabilizer and subsequently contribute to the inhibition of telomerase enzyme and cancer's proliferation [18]. It can induce DNA damage and telomere attrition by inhibiting telomerase and cell death in glioblastoma cells with minimal effects to normal cells [19]. It can also affect DNA synthesis in cancer cells. In an earlier study, thymoquinone was found to inhibit DNA synthesis, proliferation, and viability of cancerous cells, such as LNCaP, C4-B, DU145, and PC-3, but not noncancerous BPH-1 prostate epithelial cells [20].

### Thymoquinone targets carcinogenic signaling pathways

A number of carcinogenic signaling pathways or signaling molecules have been reported as thymoquinone's target. Down-regulation of androgen receptor (AR) and cell proliferation regulator E2F-1 was indicated as the mechanism behind thymoquinone's action in prostate cancer [20]. Thymoquinone and its synthetic derivative poloxin was found to inhibit the serine/threonine kinase Polo-like kinase 1 (Plk1) (usually over-expressed in many types of cancers) by interfering with its intracellular localization [21]. Another important target of thymoquinone is the signal transducer and activator of transcription 3 (STAT3) pathway. In a study, thymoquinone was found to inhibit both constitutive and interleukin-6 (IL-6)-inducible STAT3 phosphorylation, which is correlated with the inhibition of c-Src and JAK2 (Janus kinase) activation. Also, the expression of STAT3-regulated gene products, such as cyclin D1, Bcl-2, Bcl-xL, survivin, Mcl-1 and vascular endothelial growth factor (VEGF), was inhibited by thymoquinone, which ultimately increased apoptosis and killed cancer cells or inhibited their growth [22]. Suppression of STAT3 phosphorylation by thymoquinone was also found to be associated with decrease of F-actin polymerization and reduction of proliferation of human multiple myeloma cells [23]. Thymoquinone's inhibition of STAT3 phosphorylation, associated with reduction in JAK2 and c-Src activity, as well as Bcl-2, cyclin D, survivin, and VEGF was also reported in gastric cancer cells [24]. In human colon cancer cells (HCT116), thymoquinone induced apoptosis, which was associated with the up-regulation of Bax and the inhibition of Bcl-2 as well as the activation of caspases -9, -7 and -3 and the induction of the cleavage of poly-(ADP-ribose) polymerase (PARP) [25]. In molecular level, it was found that thymoquinone exerts effects by blocking STAT3 signaling via the inhibition of JAK2- and Src-mediated phosphorylation of EGFR tyrosine kinase [25]. In oral cancer cells (T28), thymoquinone showed anticancer activity via the down-regulation of proliferation activator p38 MAPK [26]. In breast cancer cell lines (MDA-MB-468 and T47D), thymoquinone interfered with PI3K/Akt signaling and promoted G(1) arrest and induced apoptosis [27]. Thymoquinone inhibited p53-mutated acute lymphoblastic leukemia (ALL) cells via the activation of a p73-dependent mitochondrial and cell cycle checkpoint signaling pathway; this pathway subsequently targets the anti-apoptotic and epigenetic integrator UHRF1, which is essential for cell cycle progression [28]. Furthermore, it was found that down-regulation of cyclic nucleotide phosphodiesterase PDE1A is the key event of p73 and UHRF1 deregulation in thymoquinone-induced ALL cell apoptosis [29]. In ALL cell line CEM-ss, thymoquinone treatment generated reactive oxygen species (ROS) and HSP70, down-regulated Bcl-2, up-regulated Bax, and activated caspase 3 and caspase 8 for inducing apoptosis [30]. A recent study confirms that thymoquinone can induce apoptosis in MCF-7 breast cancer cells via the up-regulation of p53 expression [31]. Thymoquinone significantly increased the expression of miR-34a via p53, and down-regulated Rac1 expression, followed by actin depolymerisation and disruption of the actin cytoskeleton [32]. This damage in the actin cytoskeleton leads to a significant reduction in the lamellipodia and filopodia formation on cell surfaces, thus retarding cell migration [32]. In hepatic carcinoma, thymoquinone induced cell cycle arrest and apoptosis by repressing the Notch signaling pathway [33]. A detail on the thymoquinone action in different signaling pathways involved in cancer has been reviewed by Rahmani *et al*. [34].

### Immunomodulatory activities of thymoquinone

The immunomodulatory activity of thymoquinone is another important mechanism of its anticancer activity. Evidence revealed that thymoquinone suppresses tumor necrosis factor (TNF-α)-induced NF-kappa B (NF-κB) activation, and consequently inhibits the activation of I kappa B alpha (I-κBα) kinase, I-κBα phosphorylation, I-κBα degradation, p65 phosphorylation, p65 nuclear translocation, and NF-κB -dependent reporter gene expression [35]. It also down-regulated the expression of NF-κB -regulated antiapoptotic gene products, like IAP1, IAP2, XIAP Bcl-2, Bcl-xL; surviving proliferative gene products, like cyclin D1, cyclooxygenase-2 (COX-2), and c-Myc; and angiogenic gene products like matrix metalloproteinase-9 (MMP-9) and VEGF [35]. Thymoquinone was found to suppress NF-κB signaling and IL-8 expression in childhood malignant brain tumor medulloblastoma, and it induced both extrinsic and intrinsic pathways of apoptosis [36]. It also inhibited monocyte chemo-attractant protein-1 (MCP-1), TNF-α, interleukin (IL)-1β and COX-2, ultimately reducing the NF-κB activation in pancreatic ductal adenocarcinoma cells, indicating its role as an inhibitor of proinflammatory pathways [37]. In multiple myeloma cells, thymoquinone was found to inhibit CXCL12-induced chemotaxis and increase their susceptibility to Fas-mediated apoptosis [38]. Thymoquinone is also involved in conditioning T cells *in vitro* for adoptive T-cell therapy against cancer, reported by Salem *et al*. [39], as it enhances the survival and activity of antigen-specific CD8-positive T cell. Activated B-cell lymphoma (ABC) is a subtype of diffuse large B-cell lymphoma (DLBCL), which has the worst survival rate after upfront chemotherapy, and is characterized by constitutively activated NF-κB [40]. Thymoquinone induced the release of ROS in ABC cell lines, which, in turn, inhibited NF-κB activity by dephosphorylating I-κBα and reducing the translocation of p65 subunit of NF-κB in the nuclear compartment of cells [40]. In addition to apoptosis induction, thymoquinone also plays a role in inducing autophagy in glioblastoma cells [41]. Autophagy induction was also reported in head and neck squamous cell carcinoma (HNSCC) as a result of thymoquinone treatment [42]. Thymoquinone induced caspase-independent autophagic cell death by increasing the mitochondrial outer membrane permeability and activation of JNK and p38 in CPT-11-R LoVo colon cancer cells [43]. Without affecting the tubulin levels in normal human fibroblast, thymoquinone induces degradation of α and β tubulin proteins in human astrocytoma U87 cells and in T lymphoblastic leukaemia Jurkat cells, and thus exerts anticancer activity [44]. Thymoquinone treatment in hepatic carcinoma cells (HepG2) stimulated mRNA expression of pro-apoptotic Bcl-xS and TRAIL death receptors, inhibited the expression of the anti-apoptotic gene Bcl-2, inhibited NF-κB and IL-8, and stimulated apoptosis [45]. In cholangiocarcinoma cell lines (TFK-1 and HuCCT1), thymoquinone showed anticancer activity by down-regulating PI3K/Akt and NF-κB and other regulated gene products, such as p-AKT, p65, XIAP, Bcl-2, COX-2 and VEGF [46]. Another study revealed that thymoquinone binds to oncogene PAK1, changes its conformation and scaffold function, and interferes with RAF/MEK/ERK1/2 pathway in colorectal cancer [47].

### Thymoquinone's effects on cancer cell migration and invasion

In addition to controling cancer cell proliferation, thymoquinone also reduces cancer metastasis. In human glioblastoma U-87 and CCF-STTG1 cells, thymoquinone treatment was found to influence a drastic down-regulation of Focal Adhesion Kinase (FAK), associated with a reduction of ERK phosphorylation and matrix metalloproteinase (MMP-2 and MMP-9) secretion, consequently inhibiting cell migration and invasion [48]. The immunotherapeutic and anti-metastatic role of thymoquinone in controlling and preventing metastatic melanoma has been reported in another study, where the NLRP3 inflammasome was found to be the target of thymoquinone [49]. Thymoquinone inhibited human non-small carcinoma cell lung cancer (A549 cell) proliferation and migration by reducing ERK1/2 phosphorylation [50]. It has been evident that thymoquinone treatment inhibits TWIST1 promoter activity and decreases its expression in breast cancer cell lines; leading to the inhibition of epithelial-mesenchymal transition (EMT) mediated cancer cell migration, invasion and metastasis [51]. Along with interfering with EMT, thymoquinone also attenuated mTOR activity, and inhibited PI3K/Akt signaling in bladder cancer [52, 53].

### *In vivo* success of thymoquinone as anticancer molecule

Over the last decade, a number of studies used thymoquinone against animal cancer models. Similar to *in vitro* studies, *in vivo* investigations also revealed the antioxidant activity of thymoquinone in controlling rat hepatic carcinoma by decreasing the expression of antioxidant enzymes, such as glutathione peroxidase, glutathione-s-transferase and catalase [54]. In 1,2-dimethyl-hydrazine (DMH)-induced oxidative stress during the initiation and promotion of colon carcinogenesis in rats, thymoquinone showed chemo-preventive activity by reducing oxidative stress [55]. In colorectal cancer model of mice, thymoquinone delayed the growth of tumors, reduced tumor cell invasion and increased apoptosis [56]. In a study by Yi *et al*. [57], thymoquinone was found effective in inhibiting human umbilical vein endothelial cell migration, invasion, and tube formation, thus preventing tumor angiogenesis in xenograft prostate cancer model in mice. In cellular level, suppressing the activation of Akt was indicated as mode of action [57]. In addition, thymoquionone modulates the immune system of animals, as it inhibited NF-κB expression in breast cancer model of mice and interferes with later stages of mammary tumor progression [58].

In nude mice model of human pancreatic carcinoma, thymoquinone showed anti-neoplastic and anti-metastatic effects by down-regulating MMP-9 and X-linked inhibitor of apoptosis protein (XIAP) [59]. XIAP is actually a caspase inhibitor, which was found to be down-regulated by thymoquinone also in mouse neuroblastoma cells (Neuro-2a) [60]. Degradation of XIAP and inactivation of Akt by thymoquinone has been reported in breast cancer model *in vitro* and *in vivo*, where thymoquinone exerts anti-angiogenic and anti-invasive activities [61]. In mouse xenograft model of squamous cell carcinoma, thymoquinone was found to inhibit cell proliferation and induce apoptosis by inhibiting Akt and JNK phosphorylation [62].

In mouse model of familial adenomatous polyposis (FAP), thymoquinone interfered with polyp progression by inducting tumor-cell specific apoptosis and by modulating Wnt signaling through the activation of GSK-3β, thus reducing the risk of colorectal cancer [63]. In hepatocellular carcinoma model of rat, thymoquinone showed anti-proliferative activity by regulating the G1/S phase cell cycle transition [64]. In breast cancer xenograft model of mice, thymoquinone showed anti-proliferative and pro-apoptotic effects by down-regulating p38 MAPK via the generation of ROS [65]. In triple negative breast cancer, thymoquinone reduced phosphorylation of Akt, decreased expression of XIAP, and enhanced cisplatin- and docetaxel-induced cytotoxicity [66]. Thymoquinone combined with paclitaxel showed anti-tumor activity by interplaying with the apoptosis network in triple-negative breast cancer [67]. In xenograft model of gastric cancer mice, thymoquinone showed anticancer activity by down-regulating STAT3 pathway [24].

In murine leukemic WEHI-3 cells, thymoquinone promoted natural killer of cell activities and showed highly effective cellular cytotoxicity seen in an increase of early apoptosis, up-regulation of anti-apoptotic protein Bcl2, and down-regulation of apoptotic protein Bax; this result was also implicated in WEHI-3 cell growth in the BALB/c mice [68]. In 7,12-dimethylbenz[a]anthracene (DMBA) induced breast cancer, thymoquinone treatment showed antioxidant potential and reduced MDA, LDH, ALP and AST activities and decreased the expression of Brca1, Brca2 and Id-1, consequently preventing cancer development [69]. Another *in vivo* study revealed that thymoquinone has anti-osteoclastogenic effect by inhibiting inflammation-induced activation of MAPKs, NF-κB and ROS generation followed by suppressing the gene expression of c-Fos and NFATc1 in osteoclast precursors [70]. In experimentally induced breast cancer model of mouse, thymoquinone inhibited tumor growth and cancer metastasis [51].

### Road to clinical investigation: problems and solutions

#### Current clinical trials

There is not any clinical trial for thymoquinone registered by the U.S government yet (https://clinicaltrials.gov/ct2/results?term=Thymoquinone). However, in an Arabian Phase I trial thymoquinone was found safe and well tolerated in patients upto 10 mg/kg/day, but at this dosage, there was no significant anticancer activity found [71]. The use of thymoquinone in humans is limited due to its chemical properties and poor membrane penetration capacity. Thymoquinone is chemically hydrophobic, which causes its poor solubility, and thus bioavailability. In addition, high lipophilicity of thymoquinone causes poor formulation characteristics [72]. A number of experimental studies have been conducted to overcome the pharmacokinetic problems of thymoquinone, its adverse effect. Some studies revealed that thymoquinone in combination with other chemotherapeutic drugs can show better anticancer activities, which might be an interesting option in future clinical investigation of thymoquinone.

#### Pharmacokinetic characteristics of thymoquinone

Pharmacokinetic studies showed that thymoquinone is rapidly eliminated and slowly absorbed, and hence thymoquinone has less bioavailability. The calculated absolute bioavailability of thymoquinone was reported ~58% with a lag time of ~23 min by Alkharfy *et al*. [73]. Several chemical derivatives have been used to improve the pharmacokinetic behavior of thymoquinone to increase the bioavailability. Thymoquinone-4-α-linolenoylhydrazone and thymoquinone-4-palmitoylhydrazone was found to inhibit cell proliferation dependent on p53 status by activating the cell cycle inhibitor p21 [74]. Also the development of nanoparticles has created a remarkable approach in thymoquinone delivery, which might be very effective in enhancing bioavailabity. Thymoquinone-loaded liposomes (TQ-LP) and thymoquinone loaded in liposomes modified with Triton X-100 (XLP) with diameters of about 100 nm were found to maintain stability, improve bioavailability and maintain thymoquinone's anticancer activity [72]. Encapsulation of TQ into nanoparticles with 97.5% efficiency in biodegradable nanoparticulate formulation based on poly (lactide-co-glycolide) (PLGA) and stabilizer polyethylene glycol (PEG)-5000 enhances its anti-proliferative, anti-inflammatory, and chemosensitizing effects [75]. Thymoquinone packaged in nanoparticles have been proved more useful to improve bioavailability, which is called ‘nanochemoprevention’ or ‘nano-chemotherapy’ [76]. A double mesoporous core-shell silica spheres (DMCSSs) loaded with thymoquinone was found more effective in inducing cancer cell apoptosis than free thymoquinone, due to the slow release of the drug from the mesoporous structure [77]. However, studies revealed that the aqueous solubility of thymoquinone is not a major obstacle for the drug formulations, as it possesses considerable water solubility (> 500 μg/mL), which may be enough to exert pharmacologic effects after parenteral route administration [78]. Thymoquinone-loaded nanostructured lipid carrier (TQ-NLC) has been developed to improve its bioavailability (elimination half-life ~5 hours), which can exhibit cytotociity against cancer cell lines by inducing apoptosis and cell cycle arrest [79, 80]. Bhattacharya *et al*. [32] developed thymoquinone-encapsulated nanoparticles using biodegradable, hydrophilic polymers, like polyvinylpyrrolidone and polyethyleneglycol to overcome thymoquinone's poor solubility, thermal and light sensitivity, and minimal systemic bioavailability, which can greatly improve the cancer treatment's efficiency. This nanoparticle can induce breast cancer cell killing and reduced migration. Myristic acid-chitosan (MA-chitosan) nanogels were prepared by Dehghani *et al*. [81] and thymoquinone was loaded into the nanogels for the treatment of human breast adenocarcinoma cell MCF-7. Interestingly, this nanogel was found more effective in anticancer activity than thymoquinone alone.

#### Optimum dose of thymoquinone

Another problem of clinical usage of thymoquinone lies in the safety issue. The LD50 in mice was determined 104.7 mg/kg after intra-peritoneal injection and 870.9 mg/kg after oral ingestion of thymoquionone, and in rats, LD50 was found to be 57.5 mg/kg and 794.3 mg/kg after intraperitoneal and oral ingestion respectively [82]. However, thymoquinone shows anticancer activity in very small concentrations, approximately < 10 mg/kg [51, 82]. Thus, safety issue might not pose a big problem.

#### Combination of thymoquinone with other chemotherapeutic drugs

Combination of thymoquinone with other clinically used anticancer drugs may enhance chemotherapeutic potentiality. In fact, thymoquinone has been proved to be very effective in synergistic anticancer activity with available drugs. While 5-fluorouracil is regarded as the chemotherapeutic gold-standard for certain cancers, especially colon cancer, thymoquinone's activity was found closely comparable to 5-fluorouracil in both SW-626 human colon cancer cell killing and intercellular metabolic function interference. Moreover, when used in combination with 5-fluorouracil, thymoquionone augments its apoptotic activity in gastric cancer cells *in vitro* and *in vivo* [83, 84]. A combination of thymoquionone, 5-fluorouracil, and epigallocatechin-3-gallate showed more potent anticancer activity against FaDu nasopharyngeal carcinoma cell and SK-OV-3 ovarian cancer cell line [85, 86]. A recent study reports that 5-fluorouracil and thymoquinone cooperate to repress the expression of procancerous Wnt, β-catenin, NF-κB, COX-2, iNOS, VEGF, and TBRAS; up-regulate the expression of anti-tumorigenesis DKK-1, CDNK-1A, TGF-β1, TGF-βRII, Smad4, and GPx; and show chemopreventive effects on colorectal carcinogenesis in rats [87]. Doxorubicin is another chemotherapeutic drug, whose anticancer activity is improved by combining thymoquinone in a cell-line specific manner, specifically in HL-60 and multi-drug resistant MCF-7/TOPO cancer cells [88]. Thymoquinone also induces apoptosis by up-regulating PTEN and inhibiting Akt phosphorylation in doxorubicin-resistant human breast cancer cells [89]. Cisplatin is one of the most active chemotherapeutic agents in lung cancer. Combination of cisplatin and thymoquinone is highly effective in non-small cell lung cancer (NSCLC), small cell lung cancer (SCLC) cell lines and mouse xenograft model; this combination is even able to overcome the cisplatin resistance [90]. In fact, thymoquinone was reported to be more potent than cisplatin in killing human cervical squamous carcinoma cells SiHa by inducing apoptosis with Bcl-2 down-regulation [91]. Thymoquinone enhanced cisplatin-mediated cytoxicity in ID8-NGL mouse ovarian cancer cells and ovarian cancer model C57BL/6 mice. Thymoquinone treatment actually promoted cisplatin-induced pH2AX (double-strand DNA break marker) expression in cultured cells and in tumors [92]. In multiple myeloma treatment, thymoquinone can enhance the anticancer activity of bortezomib *in vitro* and *in vivo*, and can even overcome chemoresistance [93]. Castrate-resistant prostate cancer (CRPC) is a major concern in cancer therapeutic research. The combination of thymoquione and docetaxel has been proved to be effective against CRPC cells in inducing cytotoxicity and apoptosis by modulating PI3K-Akt pathway [94]. Thymoquinone pretreatment following gemcitabine treatment synergistically increased apoptosis and inhibited tumor growth in pancreatic cancer *in vitro* and *in vivo*. This combination contributes to suppression of Notch1 and NICD accompanying with up-regulation of PTEN, inactivation of Akt/mTOR/S6 signaling pathways, and the suppression of phosphorylation and nuclear translocation of p65 induced by TNF-α. Thymoquinone pretreatment with gemcitabine therapy also down-regulated anti-apoptotic Bcl-2, Bcl-xL, and XIAP; it also up-regulated or activated the pro-apoptotic molecules, such as, Caspase-3, Caspase-9, Bax and increased release of cytochrome c [95]. Thymoquinone in combination with mesalazine reduced tumor development and multiplicity in Msh2 (loxP/loxP) Villin-Cre mice by reducing microsatellite instability independent of a functional mismatch repair system [96]. In hormone and drug resistant prostate cancer cells (PC-3 and DU-145), thymoquionone in combination with zoledronic acid showed significant synergistic cytotoxic activity and DNA fragmentation, as well as increased the caspase 3/7 activity in PC-3 cell line [97]. In combination with paclitaxel, thymoquinone was found very effective against triple negative breast cancer, both drugs worked synergistically [67]. Thymoquinone also increased the efficacy of tamoxifen in inducing apoptosis in human breast cancer cells MCF-7 and MDA-MB-231 [98]. Glioblastoma multiforme is one of the most lethal forms of human cancer, and thymoquinone was found to enhance the anticancer activity of temozolomide, which is currently part of the standard treatment for this disease [99]. A recent study showed that thymoquinone can potentiate the chemopreventive effect of vitamin D during the initiation phase of colon cancer in rat model [100]. In addition to chemotherapeutic combination, as an adjuvant, thymoquinone also mediates radiosensitization and cancer chemo-radiotherapy [101]; in combination with single dose of ionizing radiation (2.5 Gy), thymoquinone was found to exert supra-additive cytotoxic effects on MCF7 and T47D breast cancer cells by enhancing apoptosis and cell cycle modulation. Interestingly, our group recently found that thymoquinone can synergistically enhance the potential of another therapeutic agent, miR-34a [102].

#### Future direction

Considering the multiple molecular mechanism of thymoquinone action, its potency in small concentrations, *in vivo* success, enhanced bioavailability, success in combination with other drugs, it is time to focus on clinical trials for thymoquinone. Moreover, the basic biochemical or molecular biological investigations should be continued for the better understanding of the molecular mechanisms of thymoquinone. Using the nanomaterial encapsulation of thymoquinone, synthesizing its more effective new chemical derivatives with more potential pharmacokinetic characteristics might be interesting in future drug development and clinical usage. As targeting specific cancer therapeutics is a major focus in present anticancer treatment, specific molecular targets for thymoquinone, like enzymes, receptors, DNA or RNA materials should also be kept under investigation.

## CONCLUSIONS

Thymoquinone is evident as a potent anticancer molecule by regulating numerous molecular mechanisms, and it has the potential to be a good therapeutic small molecule in the prevention and treatment of cancer. Now is the right time to think about clinical trials, specifically Phase I trials. For thymoquinone delivery, it can be administered in a very low dosage encapsulated in a lipophilic biogels or nanoparticles, or be used in combination with other established chemotherapeutic drugs. Meanwhile, laboratory investigations should continue for better understanding of molecular mechanism of thymoquinone action to develop potent analogs with limited side effects and a more convenient drug delivery system, ultimately improving cancer management system.
